# Application of Erythromycin and/or *Raoultella* sp. Strain MC3 Alters the Metabolic Activity of Soil Microbial Communities as Revealed by the Community Level Physiological Profiling Approach

**DOI:** 10.3390/microorganisms8121860

**Published:** 2020-11-25

**Authors:** Mariusz Cycoń, Anna Markowicz, Tomasz J. Wąsik, Zofia Piotrowska-Seget

**Affiliations:** 1 Department of Microbiology and Virology, Faculty of Pharmaceutical Sciences, Medical University of Silesia, Jagiellońska 4, 41-200 Sosnowiec, Poland; twasik@sum.edu.pl; 2 Institute of Biology, Biotechnology and Environmental Protection, Faculty of Natural Sciences, University of Silesia, Jagiellońska 28, 40-032 Katowice, Poland; anna.markowicz@us.edu.pl (A.M.); zofia.piotrowska-seget@us.edu.pl (Z.P.-S.)

**Keywords:** erythromycin, *Raoultella* sp., catabolic activity, the community-level physiological profiling (CLPP), the resistance (RS)/resilience (RL) concept, soil microorganisms

## Abstract

Erythromycin (EM), a macrolide antibiotic, by influencing the biodiversity of microorganisms, might change the catabolic activity of the entire soil microbial community. Hence, the goal of this study was to determine the metabolic biodiversity in soil treated with EM (1 and 10 mg/kg soil) using the community-level physiological profiling (CLPP) method during a 90-day experiment. In addition, the effect of soil inoculation with antibiotic-resistant *Raoultella* sp. strain MC3 on CLPP was evaluated. The resistance and resilience concept as well as multifactorial analysis of data was exploited to interpret the outcomes obtained. EM negatively affected the metabolic microbial activity, as indicated by the values of the CLPP indices, i.e., microbial activity expressed as the average well-color development (AWCD), substrate richness (R), the Shannon–Wiener (H) and evenness (E) indices and the AWCD values for the six groups of carbon substrate present in EcoPlates until 15 days. The introduction of strain MC3 into soil increased the degradative activity of soil microorganisms in comparison with non-inoculated control. In contrast, at the consecutive sampling days, an increase in the values of the CLPP parameters was observed, especially for EM-10 + MC3-treated soil. Considering the average values of the resistance index for all of the measurement days, the resistance of the CLPP indices and the AWCD values for carbon substrate groups were categorized as follows: E > H > R > AWCD and polymers > amino acids > carbohydrates > miscellaneous > amines > carboxylic acids. The obtained results suggest a low level of resistance of soil microorganisms to EM and/or strain MC3 at the beginning of the exposure time, but the microbial community exhibited the ability to recover its initial decrease in catabolic activity over the experimental period. Despite the short-term effects, the balance of the soil ecosystem may be disturbed.

## 1. Introduction

Antibiotics, due to their common use all over the world, and continuous input and persistence in the environment, have been called emerging pollutants. Nowadays, their concentration in soils ranges from a few ng to as much as 50 mg/kg of soil [1,2]. It has been shown that antibiotics, even at very low concentrations, can generate changes in bacterial genomes and may induce the transfer of genes between individual members of microbial populations [3]. From the ecological point of view, it is important that antibiotics may adversely affect entire microbial communities, which may be illustrated by changes in their biodiversity [4,5] and metabolic activity [6,7,8].

One of the most important groups of antibiotics is macrolides, among which erythromycin (EM) has high consumption worldwide [9,10]. The activity of this antibiotic involves blocking protein biosynthesis by binding to the 50S ribosomal subunit of bacteria and is mainly directed against Gram-positive bacterial pathogens [11,12]. As a consequence of the high consumption of EM, it is being detected at various levels in wastewater and surface waters around the world [13,14,15]. Since the currently used wastewater treatment systems do not guarantee 100% removal of antibiotics, EM present in sewage sludge enters the soil, reaching a concentration of up to 72 µg/kg [16,17,18,19]. However, studies on degradation have shown that EM has relatively low stability in soil matrix, as shown by the DT50 or half-life values, and its fate strongly depends on the type of soil [20,21].

**Figure 1:**
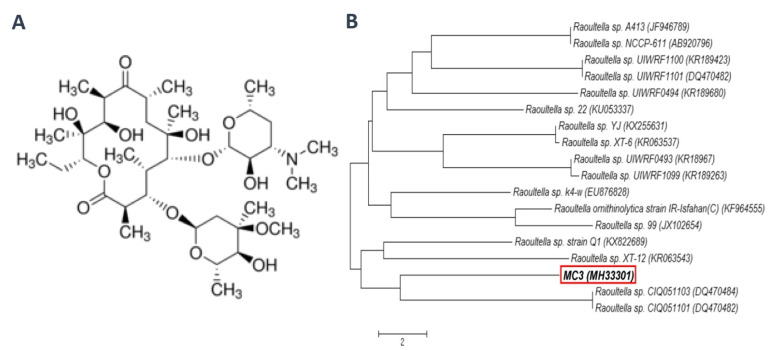
Chemical structure of erythromycin (**A**) and phylogenetic tree of *Raoultella* sp. strain MC3 (**B**).

The knowledge about the effects of EM on soil microorganisms is very limited. In our previous experiments [22], which aimed to study the effect of EM (Figure 1A) and *Raoultella* sp. strain MC3 (Figure 1B) on the genetic structure of the soil microbial community, a decrease in the biodiversity of the bacterial population up to 60 days after antibiotic application was observed. In contrast, no negative effect of strain MC3 introduced into soils on the biodiversity indices of soil microbial communities was found. However, the changes in the bacterial diversity in EM-contaminated soils may potentially disturb the functioning of soil microorganisms and thus impact the rate of ecologically important soil processes. Therefore, due to the lack of such reports, there is a need to check if the application of EM and/or *Raoultella* sp. into the soil may change the functional biodiversity of soil microorganisms. To attain such data, the community-level physiological profile (CLPP) approach and the Biolog^®^Eco™ plates were used to evaluate the catabolic potential of soil microorganisms. In addition, the resistance and resilience concept and multifactorial analysis were used to evaluate the ability of a soil microbial community to maintain its activity and functional diversity in EM-contaminated soils and/or those inoculated with *Raoultella* sp. The purpose of this study was also to indicate the usefulness of the CLPP method in measuring microbial activity, and the necessity to include it in the methodological framework for the monitoring of soil environment contaminated with antibiotics.

## 2. Materials and Methods

### 2.1. Experimental Design and Analyses

**Figure 2:**
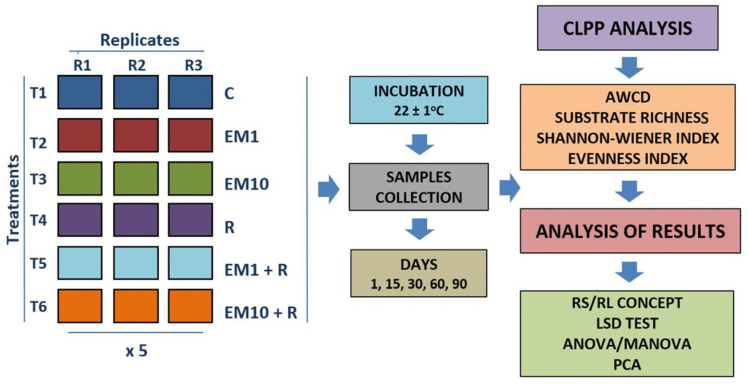
Experimental design and analyses performed. C = control. EM1 = erythromycin (1 mg/kg soil). EM10 = erythromycin (10 mg/kg soil). R = *Raoultella* sp. strain MC3. CLPP = community-level physiological profile. AWCD = average well-color development. RS = resistance. RL = resilience. LSD = least significant differences. ANOVA = analysis of variance. MANOVA = multivariate analysis of variance. PCA = principal component analysis.

The experiment was carried out in sandy loam soil [22], characterized by International Organization for Standardization (ISO) methods [23]. The experiment consisted of three replicates for the control and soil contaminated with two concentrations of EM (i.e., 1 and 10 mg/kg soil) and/or inoculation with the *Raoultella* sp. strain MC3. This strain was isolated from raw sewage using a medium supplemented with EM [22,24]. The 16S RNA gene sequencing and API 20E biochemical (bioMérieux SA, Marcy l’Etoile, France) tests identified this strain as *Raoultella* sp. [22]. The strain was designated as MC3 and its 16S rRNA gene sequence was submitted to GenBank under accession number MH333101 (Figure 1B). The resistance of *Raoultella* sp. strain MC3 to erythromycin, vancomycin, tetracycline and clindamycin determined by the MIC showed the values above 256 μg/mL [22]. In the 90-day experiment, on 1, 15, 30, 60 and 90 days, the functional diversity of the soil microbial community referred to as CLPP was assessed using the Biolog EcoPlate^TM^ (Biolog Inc., Hayward, CA, USA) [25], as detailed in the previous paper [26]. The experiment treatments and the list of parameters measured are depicted in Figure 2.

### 2.2. Analysis of the Data

The data obtained from the absorbance measurements were used to determine the CLPP indices, i.e., microbial activity expressed as the average well-color development (AWCD), substrate richness (R), and the Shannon–Wiener (H) and evenness (E) indices. The AWCD values were determined using Equation (1) [26]

AWCD = ∑OD*i*/31 (1)

where OD*_i_* is the optical density value from each well.

The H and E values were calculated using Equations (2) and (3), respectively [26],

H = −∑*pi* (ln*pi*) (2)

E = H/H*_max_*= H/lnR (3)

where *p_i_* is the ratio of the activity on each substrate (OD*_i_*) to the sum of the activities on all of the substrates (∑OD*i*), and R is the number of substrates metabolized.

The changes in the resistance (RS) and resilience (RL) of microbial communities in the soils treated with EM and/or strain MC3 at consecutive sampling days of the experiment were calculated using an approach proposed by Orwin and Wardle [27]. The RS index was calculated using Equation (4)

RS(t_0_) = 1 − 2|D_0_| / (C_0_ + |D_0_|) (4)

where D_0_ is the difference between the control (C_0_) and the disturbed soil (P_0_) at the end of the disturbance (t_0_). The RL index was calculated using Equation (5)

RL(t_x_) = 2|D_0_|/(|D_0_| + |D_x_|) − 1 (5)

where D_0_ is as above and D_x_ is the difference between the nontreated control (C_x_) and the exposed soil (P_x_) at the time point (t_x_) chosen to measure the resilience.

The data obtained for the CLPP indices (i.e., AWCD, R_S_, H and E), and the AWCD values for the six carbon substrate groups (i.e., amines, amino acids, carbohydrates, carboxylic acids, miscellaneous and polymers), were analyzed using a three-way analysis of variance (ANOVA) to assess the level of variability (%) related to the factors tested, i.e., the concentration of EM, inoculation of soil with MC3 strain and the sampling time. The data related to the RS and RL indices were subjected to two-way and one-way ANOVAs in order to examine the differences among sampling time and differences among the different treatments, respectively. The statistical significance of the differences (*p* < 0.05) was assessed using the least significant differences (LSD) test. A principal component analysis (PCA) of the data for the CLPPs and the AWCD data for the carbon substrate groups was carried out for all the sampling days and separately for each sampling day. The principal component (PC) scores from the PCA were subjected to a three-way and two-way multivariate analysis of variance (MANOVA) for the first and second PCA sets, respectively. All of the statistical analyses were done with The Statistica v. 13.0 PL software package.

## 3. Results and Discussion

Antibiotics present in the environments, due to their antimicrobial activity, may negatively influence the diversity and activity of the soil bacterial community. It has been shown that many antibiotics may decrease the overall microbial activity [26,28,29,30,31,32] and inhibit some specific process such as denitrification, nitrification, iron reduction or cumulative respiration [33,34,35].

Currently, one of the methods most commonly used to assess the functional diversity of soil microorganisms is the physiological community-level physiological profiling with the use of 96-well EcoPlates^TM^. Although CLPP does not represent the catabolic potential of the entire microbial population and limits the analysis mainly to fast-growing bacteria, it has been successfully applied to prove the negative impact of antibiotics on the metabolic activity of soil microorganisms [31,32,33].

**Figure 3:**
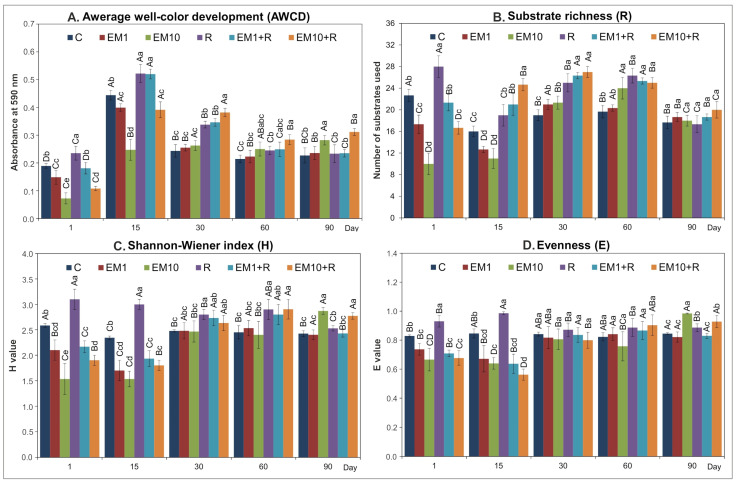
Effect of EM and/or strain MC3 on the values of the CLPP indices, i.e., AWCD (**A**), Rs (**B**), H (**C**) and E (**D**). The data presented are the means of three replicates with standard deviations. Significant differences (LSD test, *p* < 0.05) between treatments at the same sampling time and between sampling times within the same treatments are denoted with different lower and uppercase letters, respectively. The treatment abbreviations are explained in Figure 2.

The results showed that the soil inoculation with EM and/or strain MC3 altered the catabolic activity of the examined microbial community. Both doses of EM led to a significant decline (*p* < 0.05) in the values of all measured CLPP indices, i.e., AWCD (Figure 3A), R (Figure 3B), H (Figure 3C) and E (Figure 3D) till 15 days. In contrast, catabolic activity in the soil inoculated with the MC3 strain was significantly higher (*p* < 0.05) than in the control soil. In contrast, at the following sampling days, an increase in the values of the measured parameters was observed, especially for EM-10 + MC3-treated soil (Figure 3).

In general, the results of statistical analysis showed that the values of determined CLPP indices were influenced by all the analyzed factors, i.e., strain MC3, concentration of EM and incubation time, with the highest share of time in the observed variability. In addition, the interactions between the factors tested had a significant impact (Table 1). A short-term negative effect on the catabolic activity of soil microorganisms has also been noted for other antibiotics. For example, Fang et al. [36] found that AWCD and CLPP indices decreased significantly up to 35 days after chlortetracycline application. A slight inhibition on the microbial activity (expressed as the H index) in soil was observed along a gradient of oxytetracycline concentration (1–300 mg/kg soil). In contrast, a marked decline in functional diversity and AWCD values with increasing concentrations of oxytetracycline was reported by Kong et al. [31]. Antibiotics from the sulfonamide group, such as sulfamethoxazole and sulfamethazine, can also alter the activity of microbial populations; however, only short-term detrimental effects were observed [33,37]. Furthermore, sulfadimethoxine or chlortetracycline did not affect the CLPP indices including AWCD, whereas other antibiotic monensin contributed to an increase in the value of the H index [28].

**Figure 4:**
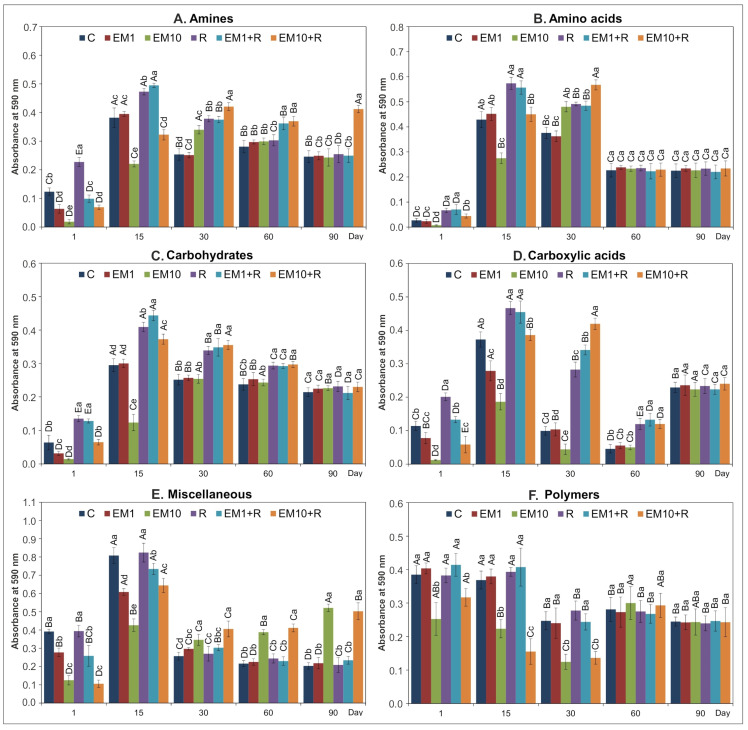
Effect of EM and/or strain MC3 on the AWCD values of carbon substrate groups, i.e., amines (**A**), amino acids (**B**), carbohydrates (**C**), carboxylic acids (**D**), miscellaneous (**E**) and polymers (**F**). The data presented are the means of three replicates with standard deviations. Significant differences (LSD test, *p* < 0.05) between treatments at the same sampling time and between sampling times within the same treatments are denoted with different lower and uppercase letters, respectively. The treatment abbreviations are explained in Figure 2.

The AWCD values demonstrated that EM and/or strain MC3 changed the pattern of carbon source utilization during the experiment (Figure 4). The introduction of EM, especially at a higher dosage, caused a significant decrease (*p* < 0.05) in the AWCD values in the case of utilization of amines, amino acids, carbohydrates and miscellaneous until 15 days and for carboxylic acids and polymers until 30 days. Furthermore, over the same time period, for soil inoculated only with strain MC3, AWCD values increased generally for all carbon substrate groups (Figure 4). On the next measurement day, i.e., on day 60, in EM and MC3 treatments, a significant increase in the catabolic activity of the analyzed microbial community for amines, carbohydrates, carboxylic acids and miscellaneous was found. Finally, at the end of the experiment, there were generally no differences between contaminated and/or inoculated soils compared to the non-treated soil (Figure 4).

In general, the results of statistical analysis demonstrated that the AWCD values of all substrate groups were affected by all analyzed factors, i.e., strain MC3, concentration of EM and time of the experiment. In addition, the interactions between above-mentioned factors had a significant impact (Table 2). In other studies, changes in the preferential degradation by microorganisms of some of the carbon substrates were observed over the course of an experimental period. For example, Xu et al. [4] revealed that sulfadiazine at a higher concentration contributed to a decrease in the utilization rates of carboxylic acids, amino acids, carbohydrates, and aromatic acids. Liu et al. [38] observed a short-term decrease in the usage of carbohydrates and miscellaneous by the microbial community in sulfamethoxazole-treated soil. In contrast, the doxycycline generally contributed to a stimulation of the substrate utilization [30].

**Figure 5:**
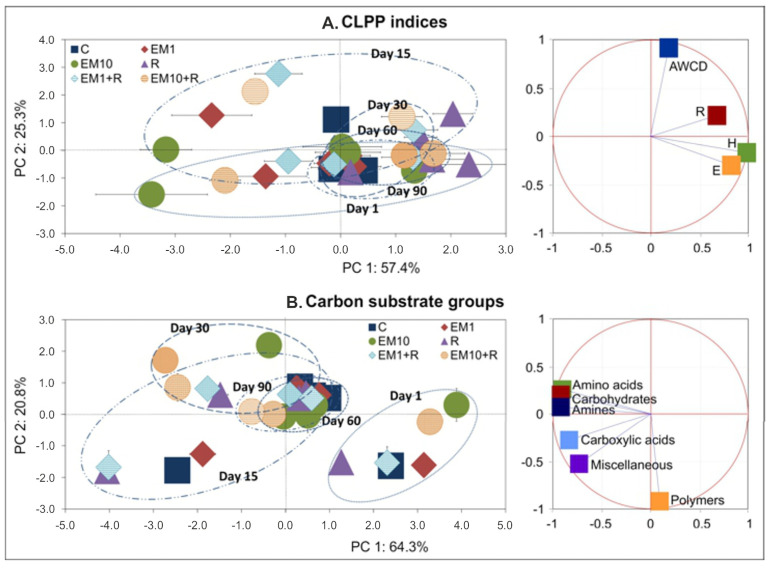
The PCA plots for the CLPP indices (**A**) and carbon substrate groups (**B**) generated for all the days of the experiment. The treatment abbreviations are explained in Figure 2.

**Figure 6:**
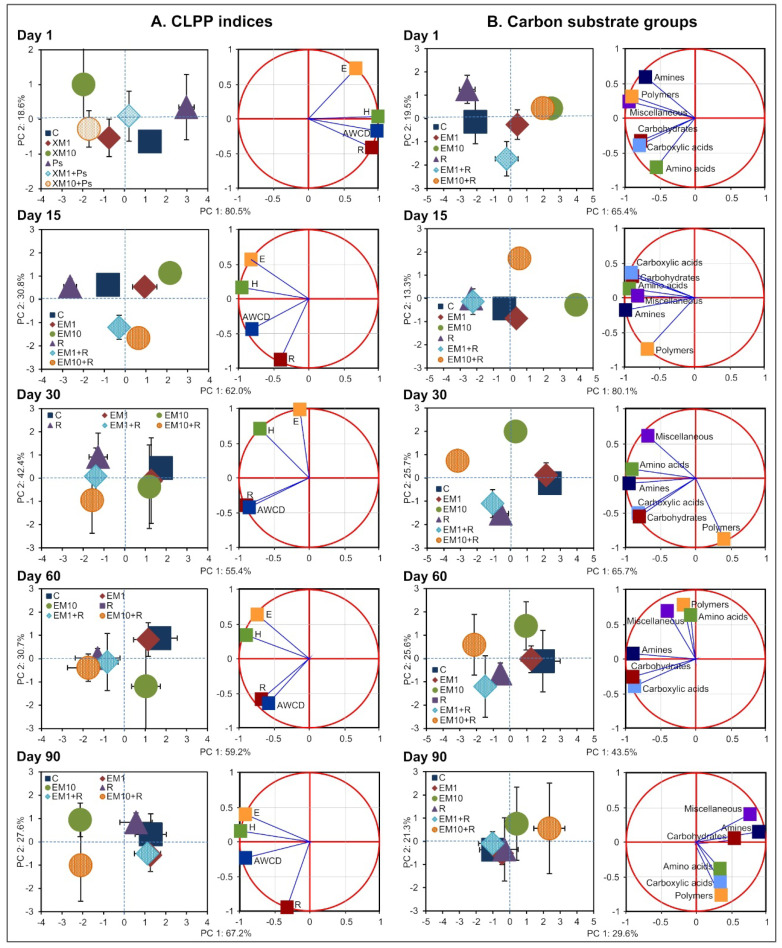
The PCA plots for the CLPP indices (**A**) and carbon substrate groups (**B**) generated for each sampling day of the experiment. The treatment abbreviations are explained in Figure 2.

The observed alterations in soil microbial functional diversity were also confirmed by PCA. The PCA plots generated for the CLPP indices (Figure 5) and the AWCD values for the carbon substrate groups (Figure 6) including all of the measurement days (Figure 5A,B) and individual sampling days (Figure 6A,B) demonstrated scattering of the samples mainly along the PC1 axis, and the three factors tested, i.e., strain MC3, the concentration of EM and time, significantly contributed to a pattern of the variability obtained (Table 3 and Table 4). Generally, an evident influence of EM concentration and/or strain MC3 on the determined parameters was observed until 30 days.

In this study, it was noted that, regardless of whether EM was used alone or in combination with strain MC3, the antibiotic contributed to environmental stress conditions, resulting in changes in the functional diversity and catabolic activity of the analyzed microbial community. The loss of the ability of the soil microbial community to utilize selected carbon substrates at the beginning of the experiment could be associated with the negative effect of EM on specific enzymes produced by microorganisms. Since EM is active mainly against Gram-positive bacteria, some members of this group of bacteria could have been killed or their metabolic activity inhibited. As a consequence of this phenomenon, catabolic activity and functional diversity significantly decreased. However, the negative effect of the antibiotic in combination with the MC3 strain observed at the beginning of the experiment was smaller compared to that observed for soil contaminated with only EM. This result suggests that part of the negative antibiotic effect was abolished by inoculation with the MC3 strain. This finding is also confirmed by the results obtained for the soil inoculated with strain MC3 alone.

Higher values of the measured parameters suggest that the introduced bacterial strain had the ability to survive in new conditions and increased the catabolic potential of the microbial community. Many studies on the degradation of pollutants in the soil environment showed synergy between inoculated strains and natural soil microflora, which resulted in the accelerated degradation of pollutants [39,40,41]. However, the lack of any effect of the introduction of bacterial strains into the soil was also observed. For example, the study by Cycoń et al. [8] showed that the antibiotic-resistant strain Citrobacter freundii did not affect the catabolic activity or functional diversity of soil microorganisms, regardless of whether it was introduced alone or in combination with vancomycin. This phenomenon may be related to the survivability of inoculants in the soil environment, which is often a foreign environment for them [39]. Soils are very complex ecosystems, and many factors, both biotic and abiotic, may determine the survival of inoculants. In addition, there may be competition between the natural soil microflora and the introduced strains of microorganisms [42]. The phenomenon of inhibition from the production of various substances by soil microorganisms that limit the activity of inoculants is also of great importance [43].

After the initial inhibition caused by EM, the values of the measured parameters increased at the subsequent sampling days. This effect could be related to the use of EM as a source of carbon and energy by selected microorganisms capable of degrading the antibiotic introduced into soil. This phenomenon could result in an increase in the number of degrading microorganisms and, hence, an increase in enzyme production. In contrast, the negative effect of EM could have been masked by the increased activity of other microorganisms capable of surviving in the presence of an antibiotic and/or using compounds released from the cells of killed microorganisms [8,44,45]. The observed effect could also be related to EM degradation in the soil. Studies on degradation have shown that EM has relatively low stability under soil conditions, as evidenced by the DT50 or half-life values of a few to several dozen days depending on the type of soil [20,21]. In addition, as has been shown by the studies of Dunkle et al. [46] and Wei et al. [47], the degradation of EM to L-cladinose, D-desosamine and a 14-member lactone ring resulted in the loss of antibacterial properties.

The observed changes in the catabolic activity of the microbial community were reflected in its resistance to stress factors (Table 5 and Table 6). In general, the results of the two-way ANOVA showed a significant impact of strain MC3, concentration of EM and time of the experiment on the resistance of the CLPP indices and the metabolic activity expressed as the AWCD for the carbon substrate groups (Table 7). Considering the average values of the resistance index for all of the measuring days, the resistance of the CLPP indices and the AWCD values for carbon substrate groups were categorized as follows: E (0.798) > H (0.744) > R (0.652) > AWCD (0.635) and polymers (0.790) > amino acids (0.647) > carbohydrates (0.604) > miscellaneous (0.545) > amines (0.509) > carboxylic acids (0.388). An analysis of the RL index for measured parameters demonstrated differences in its value for individual treatments at the end of the experiment (day 90). However, the RL index was found to be positive for most of the treated soils (Table 8).

According to the interpretation proposed by Orwin and Wardle [27], RS and RL indices may have values between −1 and 1. A value of +1 for the RS index shows the highest resistance and no effect of factors influencing the soil, while lower values show stronger effects (less resistance) related to either stimulation or inhibition. In the case of the RL index, a value of +1 at the sampling time shows maximal resilience, while lower values show a slower recovery rate. The results obtained generally suggest a low initial resistance of microorganisms to the introduction of EM and/or strain MC3, but the microbial community was resilient in the long term. Similar phenomena were noted by Cycoń et al. [9] and Baćmaga et al. [48] studying the effect of other antibiotics, i.e., vancomycin and the pesticide azoxystrobin, respectively, on the soil metabolic activity. Our results suggest that the properties and internal structure of the soil microbial community are a key factor responsible for maintaining the soil equilibrium. Despite the initial perturbations caused by stress factors, the proper balance may be restored [49,50,51]. However, the composition of the rebuilt soil community may be different to the structure of the initial community exposed to stress conditions [49,51,52].

## 4. Conclusions

It has been found out that regardless of whether EM was introduced into soil alone or in combination with *Raoultella* sp. strain MC3, it exerted stress effect on microorganisms at the beginning of the experiment, resulting in changes in the functional diversity of the soil microbial community. Interestingly, strain MC3, introduced into EM-contaminated soil, compensated for the negative effect caused by the antibiotic. Higher values of the measured parameters obtained for the soil inoculated with only the bacterial strain suggest that this strain had the ability to survive and increased the activity of soil microorganisms. It is important that after the initial inhibition of metabolic potential, its increase was noted for the subsequent measurement days. This effect could be related to an increase in the number of microorganisms that degrade antibiotics and/or masking of the effect of EM through increased activity of other microorganisms using compounds released from the cells of dead microorganisms. In addition, the degradation of EM and its loss of antibacterial properties could have great significance. In general, the results obtained suggest a low level of resistance of soil microorganisms to EM and/or strain MC3 at the beginning of the exposure time, but the microbial community had the ability to recover its initial catabolic activity over the experimental period. Despite the short-term effects, the balance of the soil ecosystem may be disturbed. The CLPP approach used in this study has shown that it may give valuable information on the metabolic activity of soil microorganisms, and despite some limitations, as in any method, it should be used for the monitoring of the soil environment affected by antibiotics.

## Figures and Tables

**Table 1:** Results of the three-way ANOVA for the CLPP indices.

SV	AWCD		R		H		E	
	VE	*p*	VE	*p*	VE	*p*	VE	*p*
S	<1	0.004 **	<1	0.403	2	<0.001 ***	3	<0.001 ***
C	11	<0.001 ***	2	<0.001 ***	5	<0.001 ***	8	<0.001 ***
T	3	<0.001 ***	38	<0.001 ***	40	<0.001 ***	25	<0.001 ***
S × C	<1	0.020 *	<1	0.586	<1	0.063	<1	0.211
S × T	<1	<0.001 ***	3	<0.001 ***	1	0.001 **	4	0.005 **
C × T	55	<0.001 ***	47	<0.001 ***	46	<0.001 ***	36	<0.001 ***
S × C × T	<1	0.866	2	0.029 *	2	0.002 **	9	<0.001 ***

SV: source of variation, S: strain, C: concentration, T: time, AWCD: average well-color development, R: substrate richness, H: Shannon–Wiener index, E: evenness index, VE: variance explained (%). Asterisks represent the significance level (* *p* < 0.05, ** *p* < 0.01 and *** *p* < 0.001).

**Table 2:** Results of the three-way ANOVA for the carbon substrate groups.

**SV**	**Amines**		**Amino Acids**		**Carbohydrates**	
	**VE**	***p***	**VE**	***p***	**VE**	***p***
S	70	<0.001 ***	<1	0.084	<1	<0.001 ***
C	<1	<0.001 ***	8	<0.001 ***	9	<0.001 ***
T	2	<0.001 ***	41	<0.001 ***	53	<0.001 ***
S × C	13	<0.001 ***	<1	<0.001 ***	<1	0.077
S × T	<1	<0.001 ***	2	<0.001 ***	1	<0.001 ***
C × T	<1	<0.001 ***	47	<0.001 ***	36	<0.001 ***
S × C × T	14	<0.001 ***	<1	<0.001 ***	<1	0.006 **
**SV**	**Carboxylic Acids**		**Miscellaneous**		**Polymers**	
	**VE**	***p***	**VE**	***p***	**VE**	***p***
S	<1	0.001 **	<1	0.002 **	<1	0.013 *
C	6	<0.001 ***	18	<0.001 ***	11	<0.001 ***
T	44	<0.001 ***	9	<0.001 ***	26	<0.001 ***
S × C	<1	0.025 *	<1	<0.001 ***	<1	0.001 **
S × T	<1	<0.001 ***	<1	0.080	1	<0.001 ***
C × T	47	<0.001 ***	72	<0.001 ***	58	<0.001 ***
S × C × T	1	<0.001 ***	<1	<0.001 ***	1	0.002 **

SV: source of variation, S: strain, C: concentration, T: time, AWCD: average well-color development, R: substrate richness, H: Shannon–Wiener index, E: evenness index, VE: variance explained (%). Asterisks represent the significance level (* *p* < 0.05, ** *p* < 0.01 and *** *p* < 0.001).

**Table 3:** Results of the three-way MANOVA for the PC 1 and PC 2 based on the CLPP indices and carbon substrate groups.

SV	CLPP Indices				Carbon Substrate Groups			
	PC 1		PC 2		PC 1		PC 2	
	VE	*p*	VE	*p*	VE	*p*	VE	*p*
S	1	<0.001 ***	1	0.072	<1	0.003 **	<1	<0.001 ***
C	8	<0.001 ***	4	0.005 **	11	<0.001 ***	2	<0.001 ***
T	35	<0.001 ***	25	<0.001 ***	34	<0.001 ***	46	<0.001 ***
S × C	<1	0.268	<1	0.456	<1	0.045 *	<1	<0.001 ***
S × T	1	0.003 **	7	0.001 **	<1	<0.001 ***	4	<0.001 ***
C × T	50	<0.001 ***	32	<0.001 ***	5	<0.001 ***	41	<0.001 ***
S × C × T	1	0.005 **	10	0.002 **	<1	0.446	4	<0.001 ***

SV: source of variation, S: strain, C: concentration, T: time, VE: variance explained (%). Asterisks represent the significance level (* *p* < 0.05, ** *p* < 0.01 and *** *p* < 0.001).

**Table 4:** Results of the two-way MANOVA for the PC1 and PC2 based on the CLPP indices and carbon substrate groups.

Day	SV	CLPP Indices				Carbon Substrate Groups			
		PC 1		PC 2		PC 1		PC 2	
		VE	*p*	VE	*p*	VE	*p*	VE	*p*
1	S	8	<0.001 ***	<1	0.758	2	0.032 *	<1	0.942
	C	86	<0.001 ***	10	0.379	94	<0.001 ***	45	0.002 **
	S × C	3	0.014 *	35	0.051	<1	0.955	31	0.007 **
15	S	5	0.009 **	<1	0.849	3	<0.001 ***	40	<0.001 ***
	C	86	<0.001 ***	4	0.725	96	<0.001 ***	33	<0.001 ***
	S × C	4	0.057	26	0.154	<1	0.080	22	<0.001 ***
30	S	11	<0.001 ***	27	0.007 **	<1	0.500	7	0.033 *
	C	81	<0.001 ***	39	0.007 **	98	<0.001 ***	33	0.001 **
	S × C	3	0.066	3	0.569	<1	0.194	47	<0.001 ***
60	S	<1	0.592	5	0.201	<1	0.438	13	0.027 *
	C	92	<0.001 ***	<1	0.959	8	<0.001 ***	47	0.002 **
	S × C	<1	0.907	66	<0.001 ***	3	0.214	16	0.050 *
90	S	<1	0.332	9	0.114	<1	0.514	59	<0.001 ***
	C	93	<0.001 ***	46	0.010 *	99	<0.001 ***	1	0.210
	S × C	1	0.334	6	0.435	<1	0.780	36	<0.001 ***

SV: source of variation, S: strain, C: concentration, VE: variance explained (%). Asterisks represent the significance level (* *p* < 0.05, ** *p* < 0.01 and *** *p* < 0.001).

**Table 5:** The resistance (RS) index for the CLPP indices.

Parameter	Day	Treatment					$\bar{x}$
		EM1	EM10	R	EM1+R	EM10+R	
**AWCD**	1	0.650^Bab^	0.237^Cc^	0.617^BCb^	0.889^Aa^	0.399^BCbc^	0.558^B^
	15	0.817^ABa^	0.386^BCb^	0.703^ABa^	0.382^Bb^	0.788^Aa^	0.615^B^
	30	0.688^ABab^	0.774^Aa^	0.438^Cbc^	0.403^Bc^	0.273^Cc^	0.515^B^
	60	0.925^Aa^	0.770^Aa^	0.907^Aa^	0.859^Aa^	0.670^ABa^	0.826^A^
	90	0.838^ABa^	0.592^ABb^	0.431^Cb^	0.863^Aa^	0.574^ABb^	0.659^B^
**Substrate richness (R)**	1	0.619^Cb^	0.283^Ec^	0.621^Bb^	0.888^Aa^	0.581^Bb^	0.598^B^
	15	0.656^Ca^	0.523^Db^	0.689^Ba^	0.527^Bb^	0.297^Dc^	0.538^B^
	30	0.814^Ba^	0.780^Ba^	0.522^Cb^	0.442^Cc^	0.407^Cc^	0.593^B^
	60	0.933^Aa^	0.641^Cb^	0.493^Cc^	0.552^Bbc^	0.573^Bb^	0.638^B^
	90	0.892^ABa^	0.962^Aa^	0.947^Aa^	0.892^Aa^	0.771^Ab^	0.893^A^
**Shannon-Wiener index (H)**	1	0.687^Ba^	0.426^Cc^	0.671^Cab^	0.722^BCa^	0.581^Db^	0.617^B^
	15	0.572^Cbc^	0.487^Cc^	0.563^Dbc^	0.704^Ca^	0.624^CDab^	0.590^B^
	30	0.929^Aa^	0.903^Aab^	0.771^Bc^	0.816^Bbc^	0.885^Aab^	0.861^A^
	60	0.935^Aa^	0.917^Aa^	0.691^BCb^	0.752^BCb^	0.688^BCb^	0.797^A^
	90	0.973^Aa^	0.689^Bb^	0.918^Aa^	0.951^Aa^	0.749^Bb^	0.856^A^
**Evenness (E)**	1	0.801^Ba^	0.676^Bb^	0.784^BCab^	0.747^Bab^	0.691^Bab^	0.740^B^
	15	0.659^Ca^	0.607^Bab^	0.716^Ca^	0.603^Cab^	0.497^Cb^	0.616^C^
	30	0.889^ABab^	0.769^ABb^	0.928^Aa^	0.938^Aa^	0.906^Aa^	0.886^A^
	60	0.957^Aa^	0.847^Aab^	0.858^ABab^	0.902^Aab^	0.826^Ab^	0.878^A^
	90	0.938^Aab^	0.717^Bc^	0.908^ABab^	0.964^Aa^	0.824^Abc^	0.870^A^

The data presented are the means of three replicates. Significant differences (LSD test, *p* < 0.05) between treatments at the same sampling time and between sampling times within the same treatments are marked with different lower and uppercase letters, respectively. A significant stimulation or inhibition in comparison to the control soil is marked in green or gray, respectively. The treatment abbreviations are explained in Figure 2.

**Table 6:** The resistance (RS) index for the carbon substrate groups.

Parameter	Day	Treatment					$\bar{x}$
		EM1	EM10	R	EM1+R	EM10+R	
**AWCD** **amines**	1	0.757^Ba^	0.281^Cc^	0.414^Cb^	0.282^Cc^	0.224^Dc^	0.392^B^
	15	0.344^Cb^	0.080^Dc^	0.086^Dc^	0.666^Aa^	0.389^Cb^	0.313^B^
	30	0.893^Aa^	0.408^Bd^	0.614^Bc^	0.542^Bc^	0.734^Ab^	0.638^A^
	60	0.941^Aa^	0.491^Bb^	0.340^Cc^	0.350^Cc^	0.205^Dd^	0.465^B^
	90	0.891^Aa^	0.876^Aa^	0.854^Aa^	0.543^Bb^	0.519^Bb^	0.736^A^
**AWCD** **amino acids**	1	0.749^Ba^	0.161^Cb^	−0.194^Cc^	−0.222^Cc^	0.219^Cb^	0.143^C^
	15	0.897^Aa^	0.472^Bb^	0.493^Bb^	0.539^Bb^	0.904^Aa^	0.661^B^
	30	0.932^Aa^	0.565^Bb^	0.530^Bb^	0.551^Bb^	0.324^Bc^	0.580^B^
	60	0.863^Ab^	0.889^Aab^	0.885^Aab^	0.963^Aa^	0.976^Aa^	0.915^A^
	90	0.886^Ab^	0.984^Aa^	0.928^Aab^	0.956^Aab^	0.925^Aab^	0.936^A^
**AWCD** **carbohydrates**	1	0.348_Bb_	0.137^Bc^	−0.068^Dd^	−0.014^Dcd^	0.738^ABa^	0.228^D^
	15	0.957_Aa_	0.263^Bd^	0.440^Cbc^	0.329^Cc^	0.582^Bb^	0.514^C^
	30	0.945_Aa_	0.978^Aa^	0.484^BCb^	0.444^Cb^	0.416^Cb^	0.653^BC^
	60	0.879_Aa_	0.945^Aa^	0.617^Bb^	0.625^Bb^	0.599^Bb^	0.733^AB^
	90	0.907_Aa_	0.893^Aa^	0.853^Aa^	0.950^Aa^	0.864^Aa^	0.893^A^
**AWCD** **carboxylic acids**	1	0.512^CDab^	0.057^Cd^	0.128^Ccd^	0.709^Ba^	0.343^Bbc^	0.350^C^
	15	0.596^Cb^	0.333^Bc^	0.596^Bb^	0.640^Bb^	0.930^Aa^	0.619^B^
	30	0.890^Aa^	0.285^BCb^	−0.301^Dc^	−0.421^Cc^	−0.529^Dc^	−0.015^C^
	60	0.306^Db^	0.777^Aa^	−0.244^Dc^	−0.318^Cc^	−0.246^Cc^	0.055^C^
	90	0.901^Aa^	0.950^Aa^	0.950^Aa^	0.952^Aa^	0.909^Aa^	0.933^A^
**AWCD** **miscellaneous**	1	0.548^Cb^	0.190^Cc^	0.934^ABa^	0.498^Cb^	0.156^Cc^	0.465^AB^
	15	0.604^Cc^	0.358^Bd^	0.961^Aa^	0.834^Ab^	0.662^Ac^	0.684^A^
	30	0.731^Bb^	0.484^Ac^	0.883^ABa^	0.693^Bb^	0.269^Bd^	0.612^AB^
	60	0.923^Aa^	0.113^Cc^	0.774^Cb^	0.886^Aa^	0.050^Dc^	0.550^AB^
	90	0.876^Aa^	−0.220^Dc^	0.853^BCa^	0.739^Bb^	−0.189^Ec^	0.412^B^
**AWCD** **polymers**	1	0.906^Aab^	0.488^Bd^	0.980^Aa^	0.860^Bb^	0.700^Bc^	0.787^AB^
	15	0.942^Aa^	0.433^BCc^	0.875^Bab^	0.821^Bb^	0.266^Cd^	0.668^B^
	30	0.887^Aa^	0.336^Cc^	0.778^Bb^	0.975^Aa^	0.380^Cc^	0.671^B^
	60	0.935^Aa^	0.881^Aa^	0.960^ABa^	0.913^ABa^	0.917^Aa^	0.921^A^
	90	0.948^Aa^	0.870^Aa^	0.939^ABa^	0.910^ABa^	0.848^Aa^	0.903^A^

The data presented are the means of three replicates. Significant differences (LSD test, *p* < 0.05) between treatments at the same sampling time and between sampling times within the same treatments are marked with different lower and uppercase letters, respectively. A significant stimulation or inhibition in comparison to the control soil is marked in green or gray, respectively. The treatment abbreviations are explained in Figure 2.

**Table 7:** Results of the two-way ANOVA for the resistance (RS) indices.

**SV/Parameter**	**AWCD**		**R**		**H**		***E***		**Amines**	
	**VE**	***p***	**VE**	***p***	**VE**	***p***	**VE**	***p***	**VE**	***p***
Tr	58	<0.01 **	50	<0.01 **	73	<0.001 ***	13	0.019 *	37	<0.01 **
T	16	<0.01 **	19	<0.01 **	3	<0.001 ***	5	0.306	15	<0.01 **
Tr × T	26	<0.01 **	28	<0.01 **	21	<0.001 ***	30	0.060	46	<0.01 **
**SV/Parameter**	**Amino acids**		**Carbohydrates**		**Carboxylic Acids**		**Miscellaneous**		**Polymers**	
	**VE**	***p***	**VE**	***p***	**VE**	***p***	**VE**	***p***	**VE**	***p***
Tr	36	<0.01 **	26	<0.01 **	60	<0.01 **	47	<0.001 ***	51	<0.01 **
T	34	<0.01 **	35	<0.01 **	14	<0.01 **	10	<0.001 ***	15	<0.01 **
Tr × T	30	<0.01 **	37	<0.01 **	24	<0.01 **	36	<0.001 ***	33	<0.01 **

SV: source of variation, Tr: treatment, T: time, AWCD: average well-color development, R: substrate richness, H: Shannon-Wiener index, E: evenness index, VE: variance explained (%). Asterisks represent the significance level (* *p* < 0.05, ** *p* < 0.01 and *** *p* < 0.001).

**Table 8:** Values of the resilience (RL) index for measured parameters obtained at the end of the experiment.

Parameter	Treatment					$\bar{x}$
	EM1	EM10	R	EM1+R	EM10+R	
AWCD	0.294^a^	0.338^a^	−0.325^b^	−0.148^ab^	0.140^ab^	0.060
Substrate richness (R_S_)	0.702^b^	0.950^a^	0.841^ab^	0.190^d^	0.450^c^	0.627
Shannon−Wiener index (H)	0.881^a^	0.391^b^	0.649^ab^	0.750^a^	0.324^b^	0.599
Evenness (E)	0.614^ab^	0.044^c^	0.441^ab^	0.791^a^	0.291^bc^	0.436
AWCD amines	0.266^ab^	0.673^a^	0.506^ab^	0.073^b^	0.104^ab^	0.324
AWCD amino acids	−0.486^c^	0.841^a^	0.654^a^	0.777^a^	0.323^b^	0.422
AWCD carbohydrates	0.457^a^	0.565^a^	0.607^a^	0.849^a^	−0.350^b^	0.426
AWCD carboxylic acids	0.516^b^	0.890^a^	0.872^a^	0.521^b^	0.658^ab^	0.691
AWCD miscellaneous	0.776^a^	−0.087^b^	−0.192^b^	0.602^a^	−0.020^b^	0.216
AWCD polymers	0.462^a^	0.775^a^	−0.248^b^	0.469^a^	0.572^a^	0.406

The data presented are the means of three replicates. Significant differences (LSD test, *p* < 0.05) between the values of each parameter are marked with different letters. The treatment abbreviations are explained in Figure 2.

## References

[1] Devries S.L., Zhang P. (2016). Antibiotics and the Terrestrial Nitrogen Cycle: A Review. Curr. Pollut. Rep..

[2] Pan M., Chu L. (2017). Leaching behavior of veterinary antibiotics in animal manure-applied soils. Sci. Total. Environ..

[3] Grenni P., Ancona V., Caracciolo A.B. (2018). Ecological effects of antibiotics on natural ecosystems: A review. Microchem. J..

[4] Xu Y., Yu W., Ma Q., Wang J., Zhou H., Jiang C. (2016). The combined effect of sulfadiazine and copper on soil microbial activity and community structure. Ecotoxicol. Environ. Saf..

[5] Orlewska K., Piotrowska-Seget Z., Cycoń M. (2018). Use of the PCR-DGGE Method for the Analysis of the Bacterial Community Structure in Soil Treated with the Cephalosporin Antibiotic Cefuroxime and/or Inoculated with a Multidrug-Resistant Pseudomonas putida Strain MC1. Front. Microbiol..

[6] Cui H., Wang S., Fu J., Zhou Z.-Q., Zhang N., Guo L. (2014). Influence of ciprofloxacin on microbial community structure and function in soils. Biol. Fertil. Soils.

[7] Liu B., Li Y., Zhang X., Wang J., Gao M. (2015). Effects of chlortetracycline on soil microbial communities: Comparisons of enzyme activities to the functional diversity via Biolog EcoPlatesTM. Eur. J. Soil Biol..

[8] Cycoń M., Orlewska K., Markowicz A., Żmijowska A., Smoleń-Dzirba J., Bratosiewicz-Wąsik J., Wąsik T.J., Piotrowska-Seget Z. (2018). Vancomycin and/or Multidrug-Resistant Citrobacter Freundii Altered the Metabolic Pattern of Soil Microbial Community. Front. Microbiol..

[9] Adriaenssens N., Coenen S., Versporten A., Muller A., Minalu G., Faes C., Vankerckhoven V., Aerts M., Hens N., On behalf of the ESAC Project Group (2011). European Surveillance of Antimicrobial Consumption (ESAC): Outpatient macrolide, lincosamide and streptogramin (MLS) use in Europe (1997–2009). J. Antimicrob. Chemother..

[10] Versporten A., Zarb P., Caniaux I., Gros M.-F., Drapier N., Miller M., Jarlier V., Nathwani D., Goossens H., Koraqi A. (2018). Antimicrobial consumption and resistance in adult hospital inpatients in 53 countries: Results of an internet-based global point prevalence survey. Lancet Glob. Health.

[11] Liang J.-H., Han X. (2013). Structure-activity relationships and mechanism of action of macrolides derived from erythromycin as antibacterial agents. Curr. Top. Med. Chem..

[12] Jelić D., Antolović R. (2016). From Erythromycin to Azithromycin and New Potential Ribosome-Binding Antimicrobials. Antibiotics.

[13] He S., Dong D., Zhang X., Sun C., Wang C., Hua X., Zhang L., Guo Z. (2018). Occurrence and ecological risk assessment of 22 emerging contaminants in the Jilin Songhua River (Northeast China). Environ. Sci. Pollut. Res..

[14] Kafaei R., Papari F., Seyedabadi M., Sahebi S., Tahmasebi R., Ahmadi M., Sorial G., Asgari G., Ramavandi B. (2018). Occurrence, distribution, and potential sources of antibiotics pollution in the water-sediment of the northern coastline of the Persian Gulf, Iran. Sci. Total. Environ..

[15] Mirzaei R., Yunesian M., Nasseri S., Gholami M., Jalilzadeh E., Shoeibi S., Mesdaghinia A. (2018). Occurrence and fate of most prescribed antibiotics in different water environments of Tehran, Iran. Sci. Total. Environ..

[16] Shi Y., Gao L., Li W., Liu J., Cai Y. (2012). Investigation of Fluoroquinolones, Sulfonamides and Macrolides in Long-Term Wastewater Irrigation Soil in Tianjin, China. Bull. Environ. Contam. Toxicol..

[17] Bin Ho Y., Zakaria M.P., Latif P.A., Saari N. (2014). Occurrence of veterinary antibiotics and progesterone in broiler manure and agricultural soil in Malaysia. Sci. Total. Environ..

[18] Pan M., Wong C.K.C., Chu L.M. (2014). Distribution of Antibiotics in Wastewater-Irrigated Soils and Their Accumulation in Vegetable Crops in the Pearl River Delta, Southern China. J. Agric. Food Chem..

[19] Gao L., Shi Y., Lihong G., Liu J., Cai Y. (2015). Occurrence and distribution of antibiotics in urban soil in Beijing and Shanghai, China. Environ. Sci. Pollut. Res..

[20] Schlüsener M.P., Bester K. (2006). Persistence of antibiotics such as macrolides, tiamulin and salinomycin in soil. Environ. Pollut..

[21] Topp E., Renaud J., Sumarah M.W., Sabourin L. (2016). Reduced persistence of the macrolide antibiotics erythromycin, clarithromycin and azithromycin in agricultural soil following several years of exposure in the field. Sci. Total. Environ..

[22] Orlewska K., Piotrowska-Seget Z., Bratosiewicz-Wąsik J., Cycoń M. (2018). Characterization of bacterial diversity in soil contaminated with the macrolide antibiotic erythromycin and/or inoculated with a multidrug-resistant Raoultella sp. strain using the PCR-DGGE approach. Appl. Soil Ecol..

[23] Cycoń M., Piotrowska-Seget Z. (2015). Biochemical and microbial soil functioning after application of the insecticide imidacloprid. J. Environ. Sci..

[24] Cycoń M., Borymski S., Orlewska K., Wąsik T.J., Piotrowska-Seget Z. (2016). An Analysis of the Effects of Vancomycin and/or Vancomycin-Resistant Citrobacter freundii Exposure on the Microbial Community Structure in Soil. Front. Microbiol..

[25] Insam H. (1997). A New Set of Substrates Proposed for Community Characterization in Environmental Samples. Microbial Communities.

[26] Orlewska K., Markowicz A., Piotrowska-Seget Z., Smoleń-Dzirba J., Cycoń M. (2018). Functional Diversity of Soil Microbial Communities in Response to the Application of Cefuroxime and/or Antibiotic-Resistant Pseudomonas putida Strain MC1. Sustainability.

[27] Orwin K., Wardle D. (2004). New indices for quantifying the resistance and resilience of soil biota to exogenous disturbances. Soil Biol. Biochem..

[28] Toth J.D., Feng Y., Dou Z. (2011). Veterinary antibiotics at environmentally relevant concentrations inhibit soil iron reduction and nitrification. Soil Biol. Biochem..

[29] Ma T., Pan X., Chen L., Liu W., Christie P., Luo Y., Wu L. (2016). Effects of different concentrations and application frequencies of oxytetracycline on soil enzyme activities and microbial community diversity. Eur. J. Soil Biol..

[30] Wang J., Lin H., Sun W., Xia Y., Ma J., Fu J., Zhang Z., Wu H., Qian M. (2016). Variations in the fate and biological effects of sulfamethoxazole, norfloxacin and doxycycline in different vegetable-soil systems following manure application. J. Hazard. Mater..

[31] Kong W.-D., Zhu Y., Fu B.-J., Marschner P., He J.-Z. (2006). The veterinary antibiotic oxytetracycline and Cu influence functional diversity of the soil microbial community. Environ. Pollut..

[32] Liu W., Pan N., Chen W., Jiao W., Wang M. (2012). Effect of veterinary oxytetracycline on functional diversity of soil microbial community. Plant Soil Environ..

[33] Pino-Otín M.R., Muñiz S., Val J., Navarro E. (2017). Effects of 18 pharmaceuticals on the physiological diversity of edaphic microorganisms. Sci. Total. Environ..

[34] Semedo M., Song B., Sparrer T., Phillips R.L. (2018). Antibiotic Effects on Microbial Communities Responsible for Denitrification and N2O Production in Grassland Soils. Front. Microbiol..

[35] Molaei A., Lakzian A., Haghnia G., Astaraei A., Rasouli-Sadaghiani M., Ceccherini M.T., Datta R. (2017). Assessment of some cultural experimental methods to study the effects of antibiotics on microbial activities in a soil: An incubation study. PLoS ONE.

[36] Fang H., Han L., Cui Y., Xue Y., Cai L., Yu Y. (2016). Changes in soil microbial community structure and function associated with degradation and resistance of carbendazim and chlortetracycline during repeated treatments. Sci. Total. Environ..

[37] Demoling L.A., Bååth E., Greve G., Wouterse M., Schmitt H. (2009). Effects of sulfamethoxazole on soil microbial communities after adding substrate. Soil Biol. Biochem..

[38] Liu F., Wu J., Ying G.-G., Luo Z., Feng H. (2011). Changes in functional diversity of soil microbial community with addition of antibiotics sulfamethoxazole and chlortetracycline. Appl. Microbiol. Biotechnol..

[39] Cycoń M., Żmijowska A., Piotrowska-Seget Z. (2014). Enhancement of deltamethrin degradation by soil bioaugmentation with two different strains of Serratia marcescens. Int. J. Environ. Sci. Technol..

[40] Hirth N., Topp E., Dörfler U., Stupperich E., Munch J.C., Schroll R. (2016). An effective bioremediation approach for enhanced microbial degradation of the veterinary antibiotic sulfamethazine in an agricultural soil. Chem. Biol. Technol. Agric..

[41] Zhang H., Zhou Y., Huang Y., Wu L., Liu X., Luo Y. (2016). Residues and risks of veterinary antibiotics in protected vegetable soils following application of different manures. Chemosphere.

[42] Chen S., Chang C., Deng Y., An S., Dong Y.H., Zhou J., Hu M., Zhong G., Zhang L.-H. (2014). Fenpropathrin Biodegradation Pathway inBacillussp. DG-02 and Its Potential for Bioremediation of Pyrethroid-Contaminated Soils. J. Agric. Food Chem..

[43] Karpouzas D.G., Walker A. (2000). Factors influencing the ability of Pseudomonas putida epI to degrade ethoprophos in soil. Soil Biol. Biochem..

[44] Ding G.-C., Radl V., Schloter-Hai B., Jechalke S., Heuer H., Smalla K., Schloter M. (2014). Dynamics of Soil Bacterial Communities in Response to Repeated Application of Manure Containing Sulfadiazine. PLoS ONE.

[45] Chessa L., Pusino A., Garau G., Mangia N.P., Pinna M.V. (2015). Soil microbial response to tetracycline in two different soils amended with cow manure. Environ. Sci. Pollut. Res..

[46] Dunkle J.A., Xiong L., Mankin A.S., Cate J.H. (2010). Structures of the Escherichia coli ribosome with antibiotics bound near the peptidyl transferase center explain spectra of drug action. Proc. Natl. Acad. Sci. USA.

[47] Wei L., Qin K., Zhao N., Noguera D.R., Qiu W., Zhao Q., Kong X., Zhang W., Kabutey F.T. (2017). Transformation of erythromycin during secondary effluent soil aquifer recharging: Removal contribution and degradation path. J. Environ. Sci..

[48] Baćmaga M., Kucharski J., Wyszkowska J. (2015). Microbial and enzymatic activity of soil contaminated with azoxystrobin. Environ. Monit. Assess..

[49] Allison S.D., Martiny J.B.H. (2008). Resistance, resilience, and redundancy in microbial communities. Proc. Natl. Acad. Sci. USA.

[50] Song H.-S., Renslow R.S., Fredrickson J.K., Lindemann S.R. (2015). Integrating ecological and engineering concepts of resilience in microbial communities. Front. Microbiol..

[51] Schäffer A., Amelung W., Hollert H., Kaestner M., Kandeler E., Kruse J., Miltner A., Ottermanns R., Pagel H., Peth S. (2016). The impact of chemical pollution on the resilience of soils under multiple stresses: A conceptual framework for future research. Sci. Total Environ..

[52] Ludwig M., Wilmes P., Schrader S. (2018). Measuring soil sustainability via soil resilience. Sci. Total. Environ..

